# Extracellular pH Modulates Neuroendocrine Prostate Cancer Cell Metabolism and Susceptibility to the Mitochondrial Inhibitor Niclosamide

**DOI:** 10.1371/journal.pone.0159675

**Published:** 2016-07-20

**Authors:** Joseph E. Ippolito, Matthew W. Brandenburg, Xia Ge, Jan R. Crowley, Kristopher M. Kirmess, Avik Som, D. Andre D’Avignon, Jeffrey M. Arbeit, Samuel Achilefu, Kevin E. Yarasheski, Jeffrey Milbrandt

**Affiliations:** 1 Mallinckrodt Institute of Radiology, Washington University School of Medicine, St. Louis, Missouri, United States of America; 2 Department of Genetics, Washington University School of Medicine, St. Louis, Missouri, United States of America; 3 Biomedical Mass Spectrometry Resource, Washington University School of Medicine, St. Louis, Missouri, United States of America; 4 Sanford Burnham Prebys Medical Discovery Institute, Orlando, Florida, United States of America; 5 Department of Surgery, Washington University School of Medicine, St. Louis, Missouri, United States of America; University of South Alabama, UNITED STATES

## Abstract

Neuroendocrine prostate cancer is a lethal variant of prostate cancer that is associated with castrate-resistant growth, metastasis, and mortality. The tumor environment of neuroendocrine prostate cancer is heterogeneous and characterized by hypoxia, necrosis, and numerous mitoses. Although acidic extracellular pH has been implicated in aggressive cancer features including metastasis and therapeutic resistance, its role in neuroendocrine prostate cancer physiology and metabolism has not yet been explored. We used the well-characterized PNEC cell line as a model to establish the effects of extracellular pH (pH 6.5, 7.4, and 8.5) on neuroendocrine prostate cancer cell metabolism. We discovered that alkalinization of extracellular pH converted cellular metabolism to a nutrient consumption-dependent state that was susceptible to glucose deprivation, glutamine deprivation, and 2-deoxyglucose (2-DG) mediated inhibition of glycolysis. Conversely, acidic pH shifted cellular metabolism toward an oxidative phosphorylation (OXPHOS)-dependent state that was susceptible to OXPHOS inhibition. Based upon this mechanistic knowledge of pH-dependent metabolism, we identified that the FDA-approved anti-helminthic niclosamide depolarized mitochondrial potential and depleted ATP levels in PNEC cells whose effects were enhanced in acidic pH. To further establish relevance of these findings, we tested the effects of extracellular pH on susceptibility to nutrient deprivation and OXPHOS inhibition in a cohort of castrate-resistant prostate cancer cell lines C4-2B, PC-3, and PC-3M. We discovered similar pH-dependent toxicity profiles among all cell lines with these treatments. These findings underscore a potential importance to acidic extracellular pH in the modulation of cell metabolism in tumors and development of an emerging paradigm that exploits the synergy of environment and therapeutic efficacy in cancer.

## Introduction

Warburg initially made the observation that cancer cells can generate energy through enhanced uptake of glucose followed by its conversion to lactate despite having adequate oxygen with which to further oxidize pyruvate in the mitochondria (Warburg effect or aerobic glycolysis) [1]. However, glucose alone is insufficient to satisfy the diverse metabolic needs of the cancer cell. Glutamine, for example, has emerged as a critical amino acid nutrient that supplies the cell with ATP for energy, contributes carbon to cellular biomass, and provides a source of nitrogen for anabolic reactions including nucleotide and hexosamine synthesis [2, 3]. Furthermore, recent evidence demonstrates that cells prefer exogenous fatty acids for membrane biosynthesis and lactate contributes to tricarboxylic acid (TCA) cycle anaplerosis [4, 5].

However, there is much evidence showing that nutrient utilization and the tumor microenvironment are closely linked. In addition to aerobic glycolysis, glucose uptake and lactate production is enhanced by hypoxia (Pasteur effect). Therefore, the synergy of the Warburg and Pasteur effects results in the excretion of lactic acid and acidification of the tumor microenvironment (pH 6.5–6.9) relative to the physiologic pH of normal tissue (pH 7.2–7.5) [6]. Thus, acidification, a hallmark of solid tumors, plays a direct role in enhancing the malignant, aggressive phenotype of cancer cells [7–11].

Acidity may not only play an important role in the enhancement of an aggressive tumor phenotype, but also may play a role in the efficacy of therapeutics that target tumors. For example, therapeutic strategies may fail as extracellular acidification can result in resistance to immunotherapy and chemotherapy [12, 13]. Therefore, a more thorough understanding of the effects of extracellular pH on cancer metabolism and physiology would facilitate the discovery of “smart” therapeutics that can synergize with the microenvironment to inhibit tumor energetics and viability.

Repeated studies both in vitro and in vivo have demonstrated that neutralization and alkalinization of acidic pH with bicarbonate can have a therapeutic effect on cancer growth [12, 14–16]. This has led to the development of novel therapeutic agents (e.g. calcium carbonate nanoparticles) that can neutralize extracellular pH and hinder tumor growth in vivo [17]. However, identification of clinically relevant pharmaceuticals that target the aggressive, treatment-resistant acidic microenvironment of tumors is desperately needed to reduce tumor burden and enhance survival.

Neuroendocrine carcinomas are a diverse array of neoplasms that arise in multiple organ systems and display a spectrum of aggressiveness from benign to metastatic [18–22]. On one end of the spectrum, “classic carcinoids” are well-differentiated, have a low index of proliferation and low rate of metastasis. Small cell carcinomas on the other hand, are poorly differentiated, have a high mitotic index, are usually disseminated at the time of diagnosis, and resistant to conventional therapy [23–25].

Neuroendocrine prostate cancer is a histologic variant of prostate cancer that is frequently associated with metastatic potential, castrate-resistant growth and therapeutic resistance [26, 27]. Moreover, androgen deprivation therapy can promote the evolution from androgen-sensitive prostate adenocarcinoma to neuroendocrine prostate cancer [28, 29]. Like high grade neuroendocrine carcinomas, neuroendocrine prostate cancer is characterized by relatively heterogeneous areas of proliferation and necrosis [30–32]. However, the role of tumor heterogeneity, specifically metabolic heterogeneity, in the development of therapeutic resistance in neuroendocrine prostate cancer has not yet been explored.

The PNEC cell line is a well characterized model for studying neuroendocrine prostate cancer [33–36]. Herein, we use this model to characterize the effects of pH on neuroendocrine prostate cancer cell metabolism. In particular, we characterize the effects of extracellular pH on PNEC cell metabolism and use mechanistic insights from this model to identify a novel therapeutic approach that is translatable across a range of castrate-resistant prostate adenocarcinoma cell lines. Our findings underscore a potential importance to acidic extracellular pH in the modulation of cell metabolism in tumors and development of an emerging paradigm that exploits the synergy of environment and therapeutic efficacy in cancer.

## Methods

### Data Analysis

All data were analyzed with GraphPad Prism. Data obtained from toxicity studies were first normalized to control or vehicle-treated groups and then plotted. To identify pH-specific effects among groups, one-way ANOVA statistical tests with a Dunnett post-test using pH 7.4 as a control group were applied. Data were plotted as the mean ± standard deviation for all groups. To ensure reproducibility, all experiments were repeated a minimum of three times with the exception of liquid chromatography studies that were repeated twice.

### Cell Culture

Cell culture media was obtained from USBiological Life Sciences (Salem, MA). PNEC monolayer cell culture was performed as previously described [34]. PNEC cells were grown in DMEM F-12 medium containing 15mM 4-(2-hydroxyethyl)-1-piperazineethanesulfonic acid (HEPES) buffer (Invitrogen) and 1.2 g/L sodium bicarbonate supplemented with 10% heat inactivated FBS, (Sigma), 4 mM glutamine, 1x non-essential amino acids (Corning), 1x B27 serum supplement (Invitrogen), 5 ng/mL epidermal growth factor (Sigma), 5 ng/ml basic fibroblast growth factor (Sigma), and 0.1% penicillin-streptomycin (Invitrogen). PC-3 cells were grown in RPMI-1640 media containing 2 g/L sodium bicarbonate supplemented with 2 mM L-glutamine, 10% heat inactivated FBS, 1% sodium pyruvate (Corning), and 0.1% penicillin-streptomycin. PC-3M cells derived from PC-3 liver metastasis in a xenograft model [37] were obtained from Dr. William Oh’s laboratory at Mount Sinai School of Medicine and were grown in DMEM F-12 media containing 15 mM HEPES and 1.2 g/L sodium bicarbonate supplemented with 2 mM glutamine, 10% heat inactivated FBS, 1x non-essential amino acids, and 0.1% penicillin-streptomycin. C4-2B cells were grown in high glucose (4.5 g/L) DMEM media containing 3.7 g/L sodium bicarbonate and supplemented with 10% heat inactivated FBS and 0.1% penicillin-streptomycin. All cells were grown to a maximum of 75% confluency. Cells were rinsed with PBS, trypsinized, neutralized with growth media, and passaged. Conventional cultureware was used for all cell lines with the exception of C4-2B cells that required poly-L-lysine coating prior to cell seeding to prevent detachment.

### Viability and Drug Toxicity Experiments

For drug toxicity experiments in all cell lines, DMEM/F-12 pH stress media was made as described previously [34]. Glucose, glutamine, and pyruvate were present in the media at standard concentrations of 25 mM, 4 mM, and 0.5 mM, respectively. pH stress media was supplemented with 10% heat inactivated FBS, 1% non-essential amino acids, and 0.1% penicillin-streptomycin. For acidic pH, 2-(N-morpholino) ethanesulfonic acid (MES), pH 6.5 was added for a final concentration of 20 mM. For physiologic pH, HEPES, pH 7.4 was added for a final concentration of 20 mM and sodium bicarbonate added for a final concentration of 0.34 g/L. For alkaline pH, tris(hydroxymethyl)aminomethane (Tris), pH 8.5 was added for a final concentration of 20 mM and sodium bicarbonate added for a final concentration of 0.34 g/L. Cells were then incubated in a humidified chamber without CO_2_ at 37°C for the duration of the experiment. Over the course of the PNEC experiments, there was minimal variation in extracellular pH, with a variation of ± 0.1 pH unit relative to the pH at the beginning of the experiment.

For nutrient deprivation pH stress experiments, DMEM/F-12 media free of glucose, glutamine, pyruvate, HEPES, and bicarbonate was supplemented with 10% heat-inactivated dialyzed FBS (Cambridge Isotope Labs), 1x non-essential amino acids, and 0.1% penicillin-streptomycin. Glucose and glutamine were supplemented as needed at 10 mM and 4 mM concentrations, respectively. 2-DG toxicity studies were conducted in the presence of 10 mM glucose. pH 6.5, 7.4, and 8.5 buffers were added to the nutrient-defined media as indicated above.

Cells were seeded into 96-well plates containing 100 μL of conventional growth media at the following densities: PNEC, 40,000 cells per well; C4-2B, 20,000 cells per well; PC-3, 15,000 cells per well; PC3-M, 15,000 cells per well. All cells were allowed to grow for 24 hours under conventional cell culture conditions. Media was then carefully aspirated with a Pasteur pipet under vacuum suction and replaced with 200 μL of the appropriate pH stress medium for either drug toxicity or nutrient deprivation studies. Drugs were added at 10 x concentrations in 20 μL volume. DMSO vehicle in the media never exceeded 0.1% final concentration. Drug toxicity and nutrient deprivation studies were conducted in a humidified chamber without CO_2_ at 37°C for 48 hours.

### Seahorse Assay

PNEC cells were seeded into a Seahorse 96 well plate at 120,000 cells per well in 200 μL of conventional growth media. Cells were incubated for one day in conventional culture conditions. On the morning of the assay, the media was then carefully aspirated with a Pasteur pipet under vacuum suction and replaced with 100 μL of pH stress media as described above. Cells were then incubated in a humidified chamber without CO_2_ at 37°C for approximately 4 hours prior to the Seahorse assay. Immediately prior to the Seahorse assay, media was carefully aspirated from each well and replaced with 180 μL of Seahorse media (prepared according to assay specifications) titrated to either pH 6.5, pH 7.4, or pH 8.5 with hydrochloric acid or sodium hydroxide. The mitochondrial stress kit was used in which oligomycin, rotenone, antimycin A, and carbonyl cyanide p-trifluoromethoxylphenyl hydrazine (FCCP) were used at a final concentration of 1 μM.

### Sulforhodamine B Cell Viability Assay

Viability was determined using the metabolism-independent sulforhodamine B assay as previously described [38]. Briefly, 100 μL 10% ice-cold trichloroacetic acid was added to each well with a multichannel pipettor and fixed overnight at 4°C. Plates were washed with room temperature water three times and dried at room temperature. 50 μL sulforhodamine B (dye added as 0.057% concentration in 1% acetic acid) was added and incubated for 30 min. Plates were then rinsed three times with 300 μL of 1% acetic acid and allowed to dry at room temperature [38]. Cells were lysed in 300 μL 10 mM Tris Base and incubated with gentle shaking for 8 hours. Plates were read in a FLUOstar Optima microplate reader (BMG Labtech). Absorbance values were measured at 530 nm.

### Mass Spectrometry Sample Preparation

Approximately 1 x 10^6^ PNEC cells were seeded in 6 well plates in 2 mL of conventional growth media and allowed to attach under conventional growth conditions. Following 24 hours, the media was replaced with pH stress media and cultured for an additional 24 hours as described above. Conditioned media was quickly pipetted from each well. Monolayers were quickly rinsed with 1 mL ice-cold PBS containing 20 mM of the MES pH 6.5, HEPES pH 7.4, or Tris pH 8.5 buffer described above, depending upon the pH of the well. PBS was quickly aspirated. For TCA cycle metabolite quantitation, 10 nmol succinic acid-d_4_ (Sigma) was added to each well as an internal standard. The plate with the cell monolayers was transferred to a bed of dry ice. 200 μL of dry ice-cold 80% methanol/20% water was added to the well, cells were removed with a cell scraper, and lysates were transferred to a microfuge tube. This was repeated two more times for a total cell extract volume of 600 μL. Samples were centrifuged at 20,000 x g for 10 minutes at 4°C to remove insoluble debris. 20 μl of ice-cold RIPA buffer (10 mM Tris pH 8.0, 1 mM EDTA, 0.5 mM EGTA, 140 mM sodium chloride, 0.1% sodium dodecylsulfate, 0.1% sodium deoxycholate, and 1% Triton X-100) containing 1 mM sodium orthovanadate and phenylmethylsulfonylfluoride was added to the insoluble protein pellet which was vortexed briefly, sonicated on ice for 5 seconds, and centrifuged at 15000 x g for 10 minutes to remove the insoluble protein pellet. Protein was quantified with the bicinchoninic acid assay (Pierce) using a bovine serum albumin standard curve. Protein quantity was used to normalize metabolite levels to cell biomass.

For liquid chromatography/mass spectrometry (LC/MS) experiments, the methanol/water extract was used without any additional manipulation. For gas chromatography/mass spectrometry (GC/MS) experiments, methanol/water extracts were dried under a stream of nitrogen gas to complete dryness. 75 μL N-Methyl-N-trimethylsilyltrifluoroacetamide in acetonitrile (1:3 ratio) was added to the vials. The reaction took place overnight at room temperature.

### GC/MS Analysis of TCA Cycle Metabolites

Derivatized samples were analyzed on a Agilent 7890A gas chromatograph interfaced to an Agilent 5975C mass spectrometer. The GC column was a HP-5MS (30 m, 0.25 mm internal diameter, 0.25 μm film coating; P.J. Cobert St. Louis, MO). A linear temperature gradient was used. The initial temperature of 80°C was held for 2 minutes and increased to 300°C at 10°/minute. The temperature was held at 300°C for 6 minutes. The samples were run in electron ionization mode and the source temperature, electron energy and emission current were 230°C, 70 eV and 300 μA, respectively. The injector and transfer line temperatures were 250°C. Selected ion monitoring was used to detect the TCA cycle metabolites. Identities of the metabolites were established from retention times and fragmentation patterns of known standards. Concentrations of TCA cycle metabolites were determined from the signal obtained from known quantities of the internal standard and normalized to protein amount.

### LC/MS Analysis of Amino Acids

5 μL of the cell extract was injected onto either a Phenomenex Kinetex hydrophilic interaction chromatography (HILIC) (150 x 4.6 mm) column or Phenomenex Synergi reverse phase (RP) (150 x 4.6mm) column. Solvents used for both HILIC and RP analyses consisted of 0.1% formic acid in water (solvent A) and 0.1% formic acid in acetonitrile (solvent B). For RP analysis, a linear gradient was used from 95% A to 5% A over 20 minutes, and holding at 5% A for five minutes. For HILIC analysis, a linear gradient was used from 5% A to 40% A over 20 minutes, and holding at 40% A for five minutes. Total analysis times for both RP and HILIC mode was 25 minutes. Flow rates for both analyses were held constant at 0.25 mL/min. The column compartment was held at 40°C for all analyses. Reference mass solution was orthogonally sprayed into the source chamber simultaneously with the sample of interest to ensure mass accuracy. Concentrations of amino acids were determined from the signal obtained from known quantities of the internal standards and normalized to protein amount.

All mass spectra were collected in positive ion mode. Gas temperature was maintained at 275°C at a flow of 12 L/min while the nebulizer pressure was held constant at 30 psig. The sheath gas temperature was 300°C at a flow of 12 L/min. The source nozzle and capillary voltages were set to 2 kV and 3 kV, respectively. The fragmenter voltage was set to 70V.

### ADP/ATP Assay

Cells were plated in a 96 well plate and incubated in pH-buffered media as described above. Following 24 hours of incubation in the appropriate media, assays were performed with the ADP/ATP Ratio Assay Kit (Sigma) according to manufacturer specifications. To determine the effects of niclosamide on ATP levels, 10 μM niclosamide was added to the wells and measured after 30 minutes of drug incubation.

### NMR Sample Preparation

Tert-butanol, EMD^™^ internal standard was purchased from Millipore. Deuterium oxide was obtained from Cambridge Isotope Laboratories. Approximately 6 x 10^6^ cells were grown in 60.1 cm^2^ dishes containing 10 to 12 mL of media, until near confluency. Following 24 hours of pH stress, 1 mL of cell conditioned media from each dish was snap frozen with dry ice. For preparation of the cell lysates, the media was aspirated and cells were washed with ice-cold PBS. The PBS was aspirated and the dish with the cell monolayer was transferred to a bed of dry ice. 300 μL of 80% methanol / 20% water was added to the well, cells removed with a cell scraper, and transferred to a microfuge tube. This was repeated two more times for a total cell extract volume of 900 μL. Samples were centrifuged at maximum speed (20,000 x g) for 10 minutes at 4°C to remove insoluble debris. Samples were lyophilized for at least 24 hours at a constant –20°C. Protein estimation on the insoluble cell pellet was performed as described above for mass spectrometry sample preparation.

The dried samples were reconstituted with 560 μL deuterium oxide with 1 mM tert-butanol as an internal standard. The sample’s pH (pD) was carefully adjusted to 7.0. The sample was centrifuged at 6,000 x g for 2 minutes to remove any particles. The homogeneous aqueous sample was loaded into 5 mm NMR tubes for NMR analysis.

For the [^13^C] NMR labeling studies, the media was prepared the same as outlined for the nutrient deprivation experiments. DMEM/F-12 media free of glucose, glutamine, pyruvate, HEPES, and bicarbonate was supplemented with 10% heat-inactivated dialyzed FBS (Cambridge Isotope Labs), 1x non-essential amino acids, and 0.1% penicillin-streptomycin. Glucose and glutamine were supplemented as needed at 10 mM and 4 mM concentrations, respectively, except that U-[13C_6_] glucose or U-[^13^C_5_,^15^N_2_] glutamine (Cambridge Isotope Labs) was added in place of the natural abundance nutrient. Buffers were then added to the media as described above for the desired pH.

### NMR Spectroscopy Analysis

NMR measurements were carried out at 25°C using a DD-II 11.75 Tesla spectrometer (Agilent/Varian) equipped with a reverse-detection probe. Two NMR measurements were executed for all samples. The first was a 16-transient CPMG (Carr-Purcell-Meiboom-Gill) with water presaturation and ^13^C decoupling (where proton doublets bounded to ^13^C are collapsed to singlets at a chemical shift identical to protons bound to ^12^C) under quantitative equilibrium conditions. The sweep width was 6983 Hz and the preacquisition delay was 18 s. For cell growth media the 90° pulse width was 9.4–9.8 μs and for cell extraction the 90° pulse width was 8.3–8.6 μs empirically determined based upon sample concentration. Spectra were processed with an exponential apodization function corresponding to 1 Hz line-broadening factor and zero-filling to 64K. Substrate and metabolite concentrations were determined from the CPMG data through prior calibration relative to 1 mM tert-butanol established by equilibrium pulse and collect conditions.

The second NMR measurement was a first increment gHSQC (gradient heteronuclear single quantum coherence spectroscopy) experiment to effectively measure only the [^13^C]-enrichment of substrates and metabolites. This acquisition was collected for 128 transients under quantitative equilibrium conditions similar to the CPMG sequence with a 6983 Hz sweep width, 18 s preacquisition delay and a [^13^C]-decoupling 90° pulse width of 12 μs. The same 90° pulse width was used as in the CPMG measurements of the growth media and cell extraction samples. The [^13^C]-enrichment was calibrated with regular and U-^13^C-labeled glucose, glutamine, and lactate standards using similar parameters as above. The free induction decay values were multiplied by a Gaussian apodization function with a 0.1 s time constant. The relative amplitude of substrates or metabolites was calculated with Agilent CRAFT (Complete Reduction to Amplitude-Frequency Table) software incorporated in VnmrJ4.2 (Agilent).

### Mitochondrial Potential Indicator Measurements

Following cell incubation at appropriate pH for 24 hours in a 96 well plate, the mitochondrial potential indicator tetramethylrhodamine methyl ester perchlorate (TMRM; Molecular Probes) in DMSO and nuclear dye Hoechst 33342 was added to wells at a final concentration of 10 nM and 1 μg/mL respectively and incubated under conventional conditions for 1 hour. Media containing dye was carefully replaced with fresh media. The Operetta High Content Imaging System (Perkin Elmer) was then used to image the cells using 520-550/590-640 nm ex/em filters for TMRM and 360-400/410-480 nm ex/em filters for Hoechst. Fluorescence of both dyes was imaged for 200 ms. During imaging, cells were maintained at 37°C with no CO_2_. For TMRM kinetic assays, niclosamide was added to the wells with a multichannel pipette at a 10 μM final concentration and sequential imaging was performed every minute for 30 minutes. Data was exported and analyzed with ImageJ. Regions of interest were drawn around entire cells to determine TMRM intensity. For kinetic assays, the intensity of each region of interest was measured over the course of 30 minutes and plotted.

### Confocal Microscopy of Mitochondria

PNEC cells were grown on LabTek microscope slides and incubated in pH stress media for 24 hour as described above. MitoTracker Green (ThermoFisher) was added to the dishes at a final concentration of 20 nM and incubated for 1 hr. Labeling media was replaced with fresh pH stress media and imaged with an Olympus FV1000 microscope using a 488 nm laser for excitation. Z-stacked images through the cells were constructed to visualize mitochondrial morphology.

## Results

### Global profiling of PNEC metabolism

Previously, we identified that shifting extracellular pH 1 unit above (pH 8.5) or below (pH 6.5) physiologic pH modulated activity of the gamma-aminobutyric acid (GABA) shunt of the TCA cycle in PNEC cells [34]. We hypothesized that extracellular pH would have global effects on cellular metabolism. We used high resolution NMR spectroscopy to profile global changes in PNEC metabolism when incubated in an acidic pH of 6.5 and alkaline pH of 8.5 relative to a physiologic pH of 7.4.

**Fig 1:**
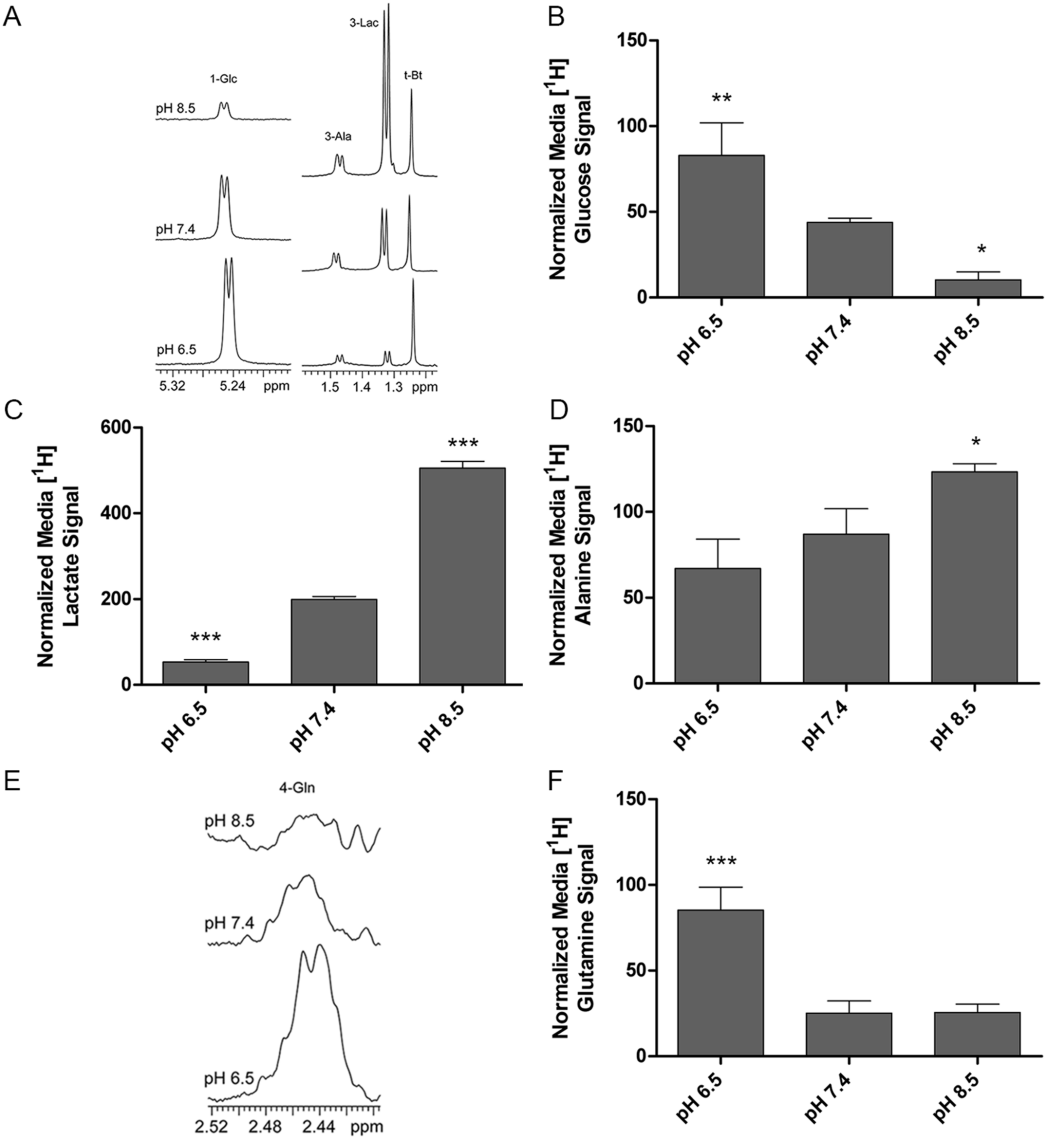
Increasing extracellular pH enhances nutrient uptake and metabolism in PNEC cells. (A) [^1^H] NMR analysis of conditioned media demonstrating increased glucose (Glc) consumption and metabolism with alkalinization. (B-D) NMR-based quantification of glucose consumption, alanine (Ala) production and lactate (Lac) production with alkalinization. (E) [^1^H] NMR analysis of conditioned media demonstrating increased glutamine consumption with alkalinization. (F) NMR-based quantification of glutamine consumption. Statistical comparisons performed relative to pH 7.4 in each group. N = 3 samples per group. *p<0.05, **p>0.01, ***p<0.001. t-Bt: t-butanol standard.

NMR spectroscopy of conditioned media following incubation at pH 6.5, 7.4, and 8.5 for 24 hours revealed that a shift toward alkaline pH depleted glucose within the media with concomitant increases in lactate and alanine (Fig 1A). These findings are consistent with enhanced glucose metabolism where pyruvate, an endpoint in glycolysis can be metabolized into lactate and alanine in addition to further metabolism in the TCA cycle [39, 40]. Quantification of the NMR signals revealed an 8.3-fold decrease in media glucose levels at pH 8.5 relative to pH 6.5 (Fig 1B). Lactate was significantly enriched 9.5-fold at pH 8.5 relative to pH 6.5 (Fig 1C). Alanine was also significantly enriched 1.9-fold at pH 8.5 relative to pH 6.5 (Fig 1D). We then determined if consumption of other nutrients was enhanced with alkalinization. Glutamine, for example, is a critical nutrient source for cancer cells [2, 3]. Glutamine levels, similarly to glucose, decreased in conditioned media as a function of alkalinization (Fig 1E). Glutamine consumption showed a trend similar to glucose with a 3.4-fold decrease in media from pH 6.5 to pH 8.5 (Fig 1F). Together, our findings supported the possibility that alkalinization of extracellular pH resulted in a concomitant shift in cellular metabolism toward enhanced nutrient uptake and metabolism.

**Fig 2:**
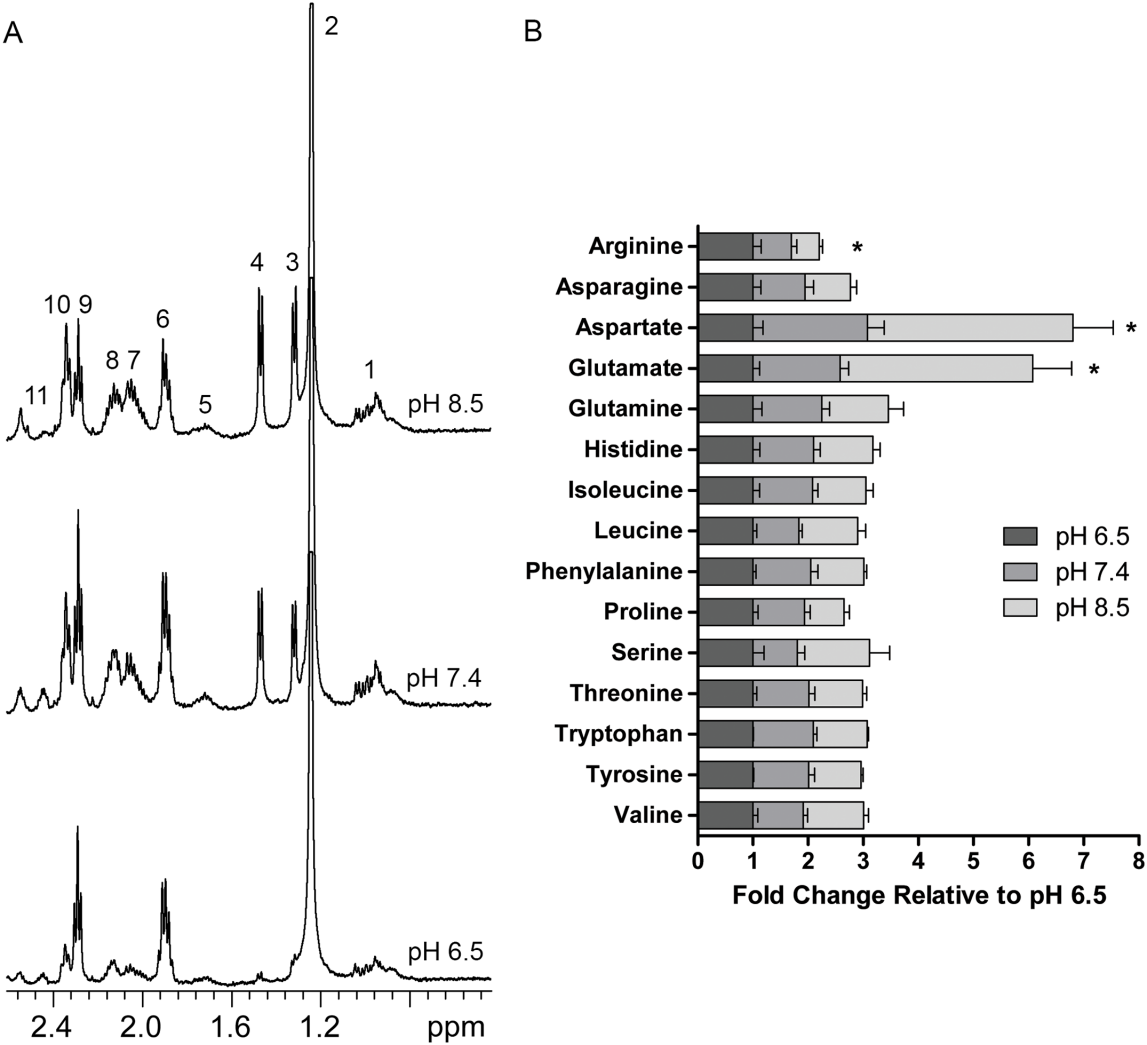
Increasing extracellular pH enhances selected amino acid levels in PNEC cells. (A) NMR of PNEC cell lysates identifies increases in alanine and glutamate and decrease in GABA. 1, valine/leucine/isoleucine; 2, t-butanol (internal reference); 3, lactate; 4, alanine; 5, lysine; 6 and 9, gamma-aminobutyric acid (GABA); 7 and 10, glutamate; 8 and 11, glutamine and reduced glutathione (GSH)/oxidized glutathione (GSSG). (B) Targeted LC/MS quantitation of selected amino acids reveals significant increases in aspartate and glutamate levels and a significant decrease in arginine levels in PNEC cells as a function of alkalinization. Statistical comparisons performed relative to pH 7.4 for each metabolite. N = 6 samples per group. *p<0.05.

Given that alkalinization enhanced amino acid uptake (*i*.*e*. glutamine) and amino acid production (*i*.*e*. alanine), we used NMR of PNEC cell lysates to determine if quantities of additional amino acids were altered. We identified that alanine levels in PNEC cells (~1.5 ppm, Fig 2A) increased with alkalinization, mirroring the trends seen in conditioned media. We also identified that glutamate paralleled alanine levels in cell lysates, increasing as function of extracellular alkalinization (Fig 2A), thus supporting our previous results [34].

These findings prompted us to determine if there were other amino acids whose concentration varied with extracellular pH. We used LC/MS to quantify changes in amino acid levels in PNEC cell lysates as a function of extracellular pH. We identified three amino acids with significant changes. Of the amino acids profiled in this study, arginine was the sole amino acid whose levels were inversely proportional to extracellular pH with a significant decrease at pH 7.4 (0.7-fold relative to pH 6.5) followed by a 0.5-fold change at pH 8.5 relative to pH 6.5. Conversely, glutamate and aspartate levels significantly increased with alkalinization with a 3.5-fold increase and 3.7-fold increase respectively at pH 8.5 (Fig 2B). Interestingly, the other amino acids were statistically unchanged between the different pH treatments. However, the concomitant enrichment of alanine, a transamination product of pyruvate, as well as glutamate and aspartate, transamination products of the TCA cycle metabolites oxaloacetate and alpha-ketoglutarate respectively, suggested that TCA cycle flux might also be affected by extracellular pH.

### Effects of pH on glycolytic and glutaminolytic flux

**Fig 3:**
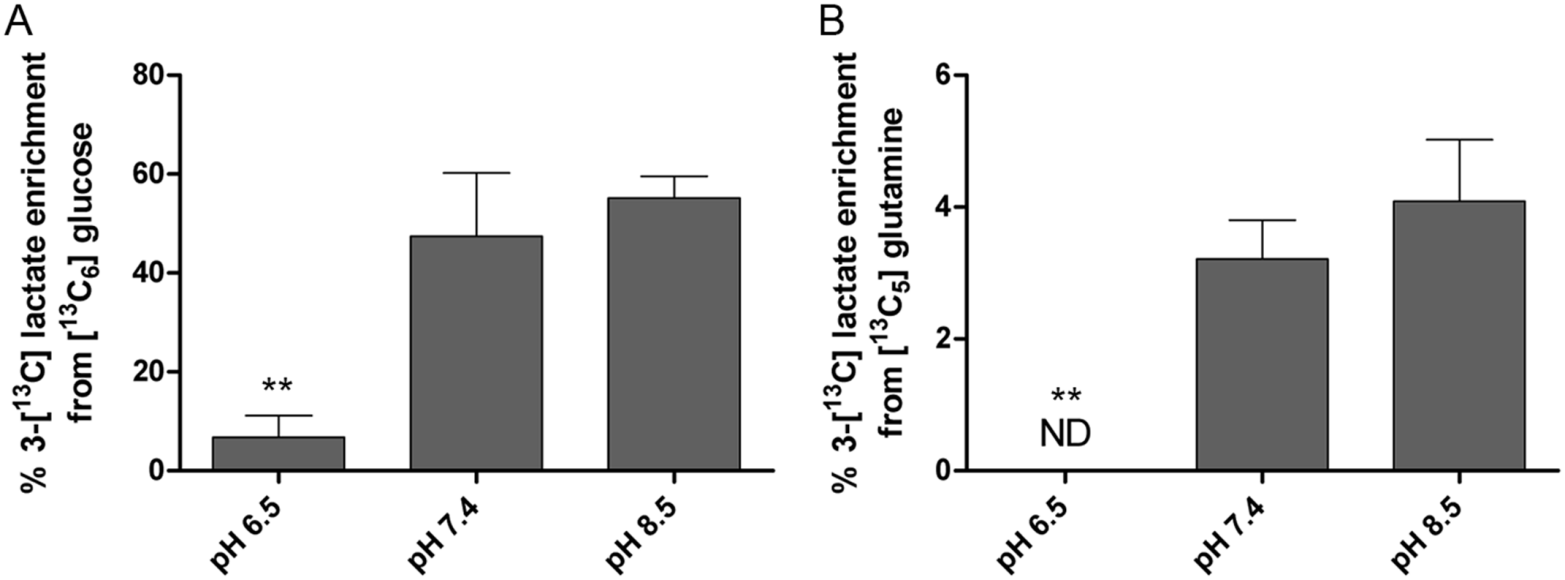
Lactate derived from glucose and glutamine increase with increasing extracellular pH. Extracellular lactate measured in the C3 position from (A) ^13^C_6_ glucose and (B) ^13^C_5_ glutamine significantly increases from pH 6.5 to pH 7.4 and demonstrates an increasing trend from pH 7.4 to H 8.5. Statistical comparisons performed relative to pH 7.4. N = 3 samples per group. **p<0.01. ND: not detected.

To address the possibility that extracellular pH could modulate both glycolysis and the TCA cycle, we investigated the role of each of these pathways in lactate synthesis. Although we assumed that lactate production was a function of enhanced glucose consumption, the possibility remained that lactate could also be produced from metabolism of glutamine (glutaminolysis) into TCA cycle intermediates, followed by conversion of malate to pyruvate via the malate aspartate shuttle and the conversion of pyruvate to lactate via lactate dehydrogenase (LDH) [41]. To quantify the relative contributions of glucose and glutamine to lactate production, we incubated PNEC cells in the presence of stable isotope labeled precursors [^13^C_6_] glucose and [^13^C_5_] glutamine at pH 6.5, 7.4, and 8.5 for 24 hours. Assays of [^13^C] lactate in conditioned media following incubation identified that lactate was generated from both glycolysis and glutaminolysis and was enhanced with alkalinization. Interestingly, the change from pH 6.5 to 7.4 resulted in the largest significant increase in [^13^C] lactate production via glycolysis from 7% to 47% [^13^C] lactate labeling (Fig 3A). Moreover, interrogation of [^13^C] glutamine metabolism to [^13^C] lactate disclosed a similar trend. Although the overall magnitude of [^13^C] lactate labeling from glutaminolysis was smaller than the labeling from glycolysis, there was a statistically significant increase in lactate production from glutamine when pH was increased from 6.5 (no [^13^C] lactate production) to 7.4 where 3.2% of lactate produced contained [^13^C] from glutamine (Fig 3B). Together, these findings demonstrated that the majority of lactate produced in PNEC cells came from glycolysis, with smaller contributions from glutamine as extracellular pH increased.

### Effects of Extracellular pH on the TCA Cycle

**Fig 4:**
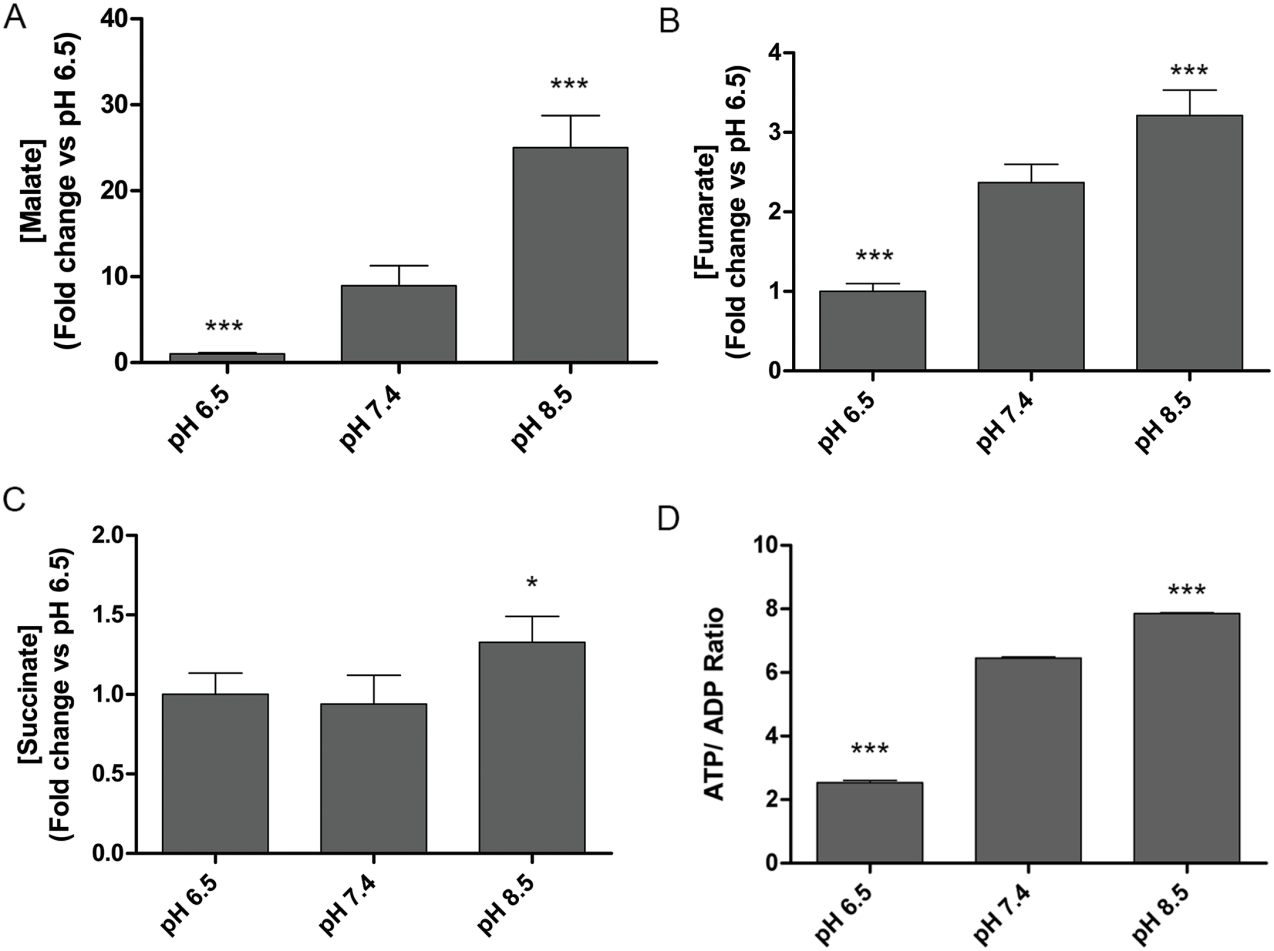
Extracellular pH modulates TCA cycle metabolism. Targeted GC/MS quantification of TCA cycle metabolites (A) malate, (B) fumarate, and (C) succinate in PNEC cells demonstrate that TCA cycle metabolite levels increase with alkalinization. (D) The ATP / ADP ratio, a marker for cellular energetics also increases with alkalinization. Statistical comparisons performed relative to pH 7.4 for each metabolite. N = 6 samples per group. *p<0.05, **p>0.01, ***p<0.001.

Aerobic glycolysis describes the generation of lactate from glycolysis with comparatively low flux of glucose into the TCA cycle despite the presence of oxygen for respiration [1]. Glutaminolysis, on the other hand, describes the enhancement of glutamine metabolism into the TCA cycle and the resultant production of lactate [41]. We determined the effects of extracellular pH on the TCA cycle through GC/MS-based quantification of malate, fumarate, and succinate. In each case, we identified statistically-significant increases of these metabolites that directly correlated with extracellular pH. Malate and fumarate were enriched under alkaline conditions by 25.0 and 3.2-fold respectively (Fig 4A and 4B). Despite robust increases in these TCA cycle metabolites; however, there was only a modest but significant increase in succinate (1.3-fold; Fig 4C). This finding was intriguing as succinate is not only a substrate for succinate dehydrogenase (SDH; complex II of OXPHOS), but is also the end product of the GABA shunt [33]. This finding supported our previous observation that GABA levels were decreased in alkaline pH [34]. It also suggested that at least part of the succinate pool in PNEC cells could be derived from the GABA shunt. Moreover, the intersection of succinate metabolism by SDH/complex II of OXPHOS and the reliance of OXPHOS on the proton motive force within the inner mitochondrial membrane suggested that extracellular pH could have effects on mitochondrial potential and OXPHOS activity. Collectively, these findings were also supported by significantly enriched ATP/ADP levels in PNEC cells that directly correlated with extracellular pH (Fig 4D). This supported our metabolic model where the combination of enhanced aerobic glycolysis and TCA cycle metabolism provided more ATP in an alkaline environment. These data also supported our findings that alkalinization enhanced glutamate and aspartate levels that are derived directly from TCA cycle metabolites.

### Effects on extracellular pH on mitochondria

**Fig 5:**
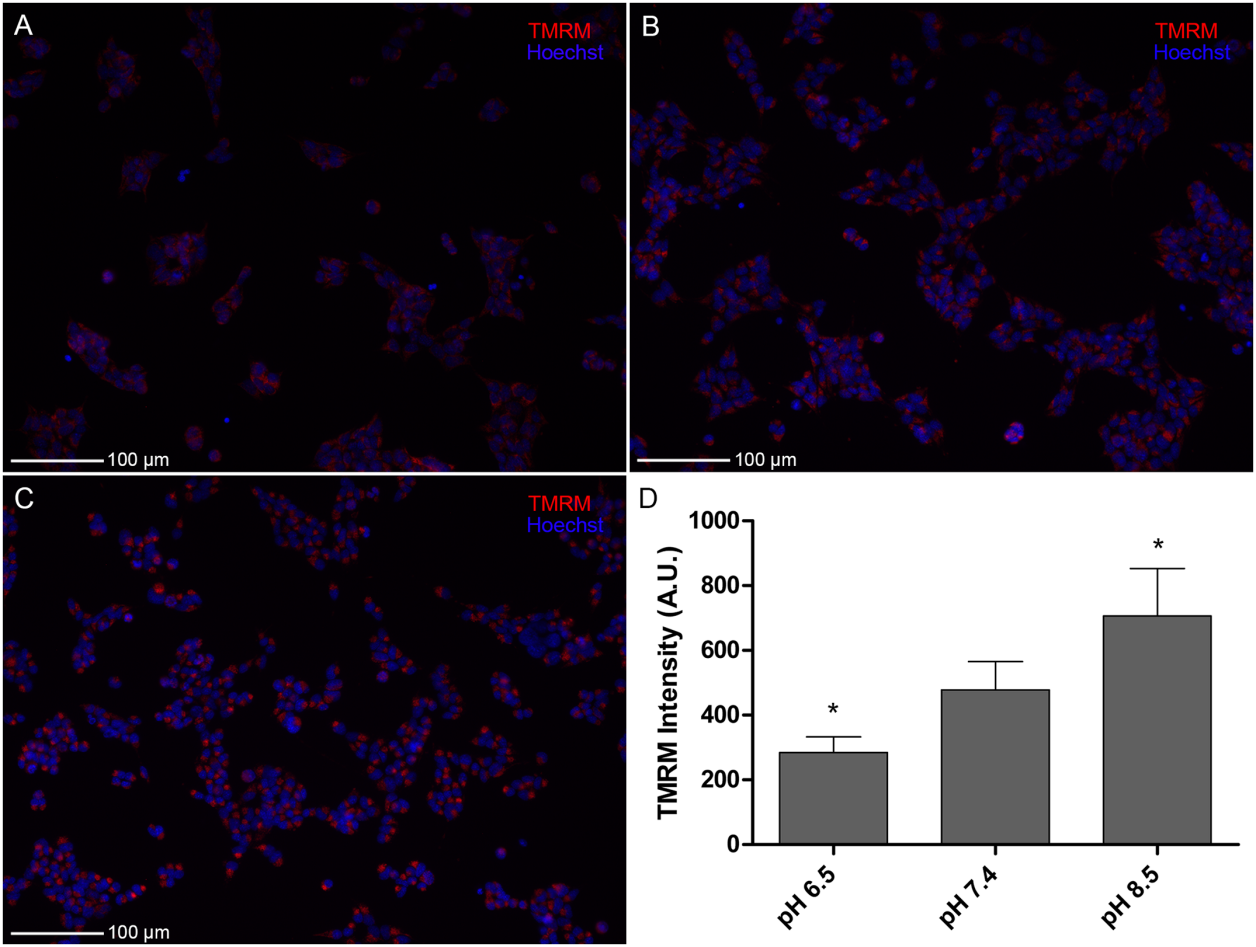
Extracellular pH modulates mitochondrial potential. Tetramethylrhodamine (TMRM) staining of PNEC cells following 24 hours of incubation at given pH values demonstrate increasing mitochondrial potential with increasing extracellular pH. (A-C) Photomicrographs of TMRM (red) staining of PNEC cells (Hoechst nuclear stain = blue). (D) Quantified TMRM intensity as a function of pH. Statistical comparisons for TMRM performed relative to pH 7.4. N = 10 samples per group. *p<0.05. A.U.: arbitrary units.

To assess the effects of extracellular pH on mitochondrial function, we applied TMRM, a fluorescent probe to measure mitochondrial potential, to PNEC cells cultured at pH 6.5, 7.4, and 8.5 for 24 hours. We identified that alkalinization of extracellular pH enhanced TMRM intensity in PNEC cells (Fig 5A–5C). Quantification of TMRM intensity disclosed a 1.7-fold increase in TMRM intensity from pH 6.5 to 7.4 and 2.5-fold increase from pH 6.5 to pH 8.5 (Fig 5D).

**Fig 6:**
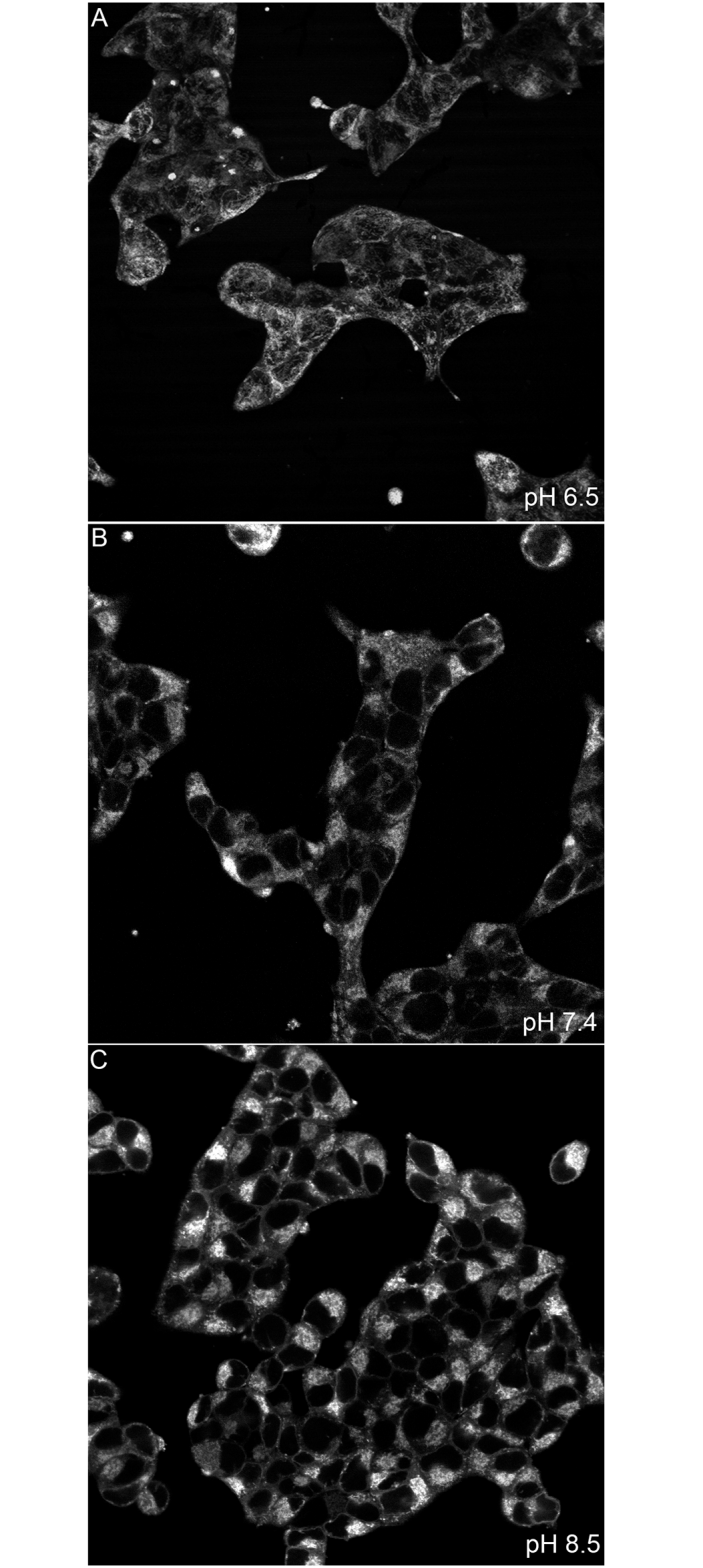
Extracellular pH modulates mitochondrial morphology in PNEC cells. Confocal imaging of PNEC cells stained with mitotracker green following 24 hours of incubation at given pH values demonstrate increasing aggregation of mitochondria with alkalinization. (A) pH 6.5, (B) pH 7.4, (C) pH 8.5.

Interestingly, we also identified a qualitative alteration in mitochondrial morphology that changed with extracellular pH. At pH 6.5, mitochondrial staining was diffusely distributed throughout the cell body (Fig 6A). However, the transition to pH 7.4 and pH 8.5 resulted in mitochondrial aggregation (Fig 6B and 6C). Together, this supported our findings that extracellular pH could modulate mitochondrial metabolism and suggested that extracellular pH could modulate the cell’s dependence on specific energetic pathways.

**Fig 7:**
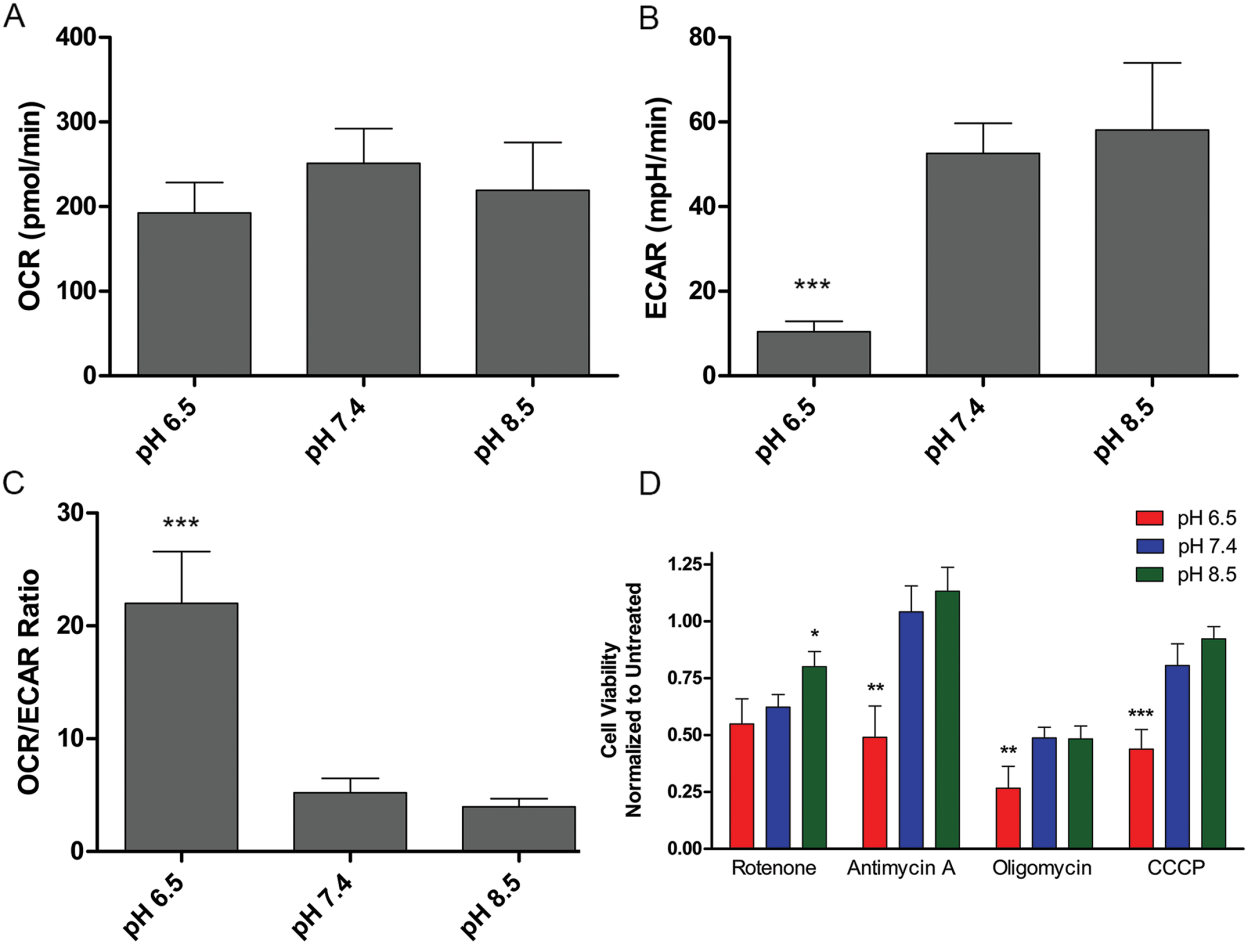
Acidity promotes an OXPHOS-dependent state in PNEC cells. (A-C) Seahorse analysis of PNEC metabolism as a function of extracellular pH. (D) OXPHOS inhibitor toxicity studies in PNEC cells as a function of extracellular pH. Statistical comparisons performed relative to pH 7.4 in each group. N = 10 samples per group for Seahorse studies, N = 4 samples per group for toxicity studies. *p<0.05, **p<0.01, ***p<0.001. OCR: Oxygen Consumption Rate, ECAR: Extracellular Acidification Rate.

Next, we investigated the effects of extracellular pH on OXPHOS activity. Interestingly, there were no differences in basal oxygen consumption rate (OCR) between PNEC cells cultured at pH 6.5, 7.4, or 8.5 (Fig 7A). However, there was enhancement of the extracellular acidification rate (ECAR) with alkalinization (Fig 7B), further supporting our findings that lactate production from both glucose and glutamine metabolism increased as extracellular pH increased. The change in ECAR coupled with constant OCR resulted in an OCR/ECAR ratio that decreased with alkalinization (Fig 7C). Together, these results signified two important findings: (i) PNEC cells cultured in physiologic and alkaline pH could derive energy from both glycolysis and OXPHOS and (ii) PNEC cells cultured in acidic pH were dependent primarily upon OXPHOS. The dependence of PNEC cells on OXPHOS in acidic pH suggested that this could be used as a therapeutic strategy.

We tested the hypothesis that OXPHOS inhibition synergizes with acidic extracellular pH to enhance PNEC toxicity. Following a two day inhibitor challenge, we identified pH-dependent cell toxicity of these inhibitors with greatest effects at pH 6.5. Interestingly, antimycin A and carbonyl cyanide m-chlorophenyl hydrazine (CCCP) had significant toxic effects on PNEC cells at pH 6.5 where, 49% of antimycin A-treated PNEC cells and 44% of CCCP-treated PNEC cells were viable relative to vehicle-treated cells (Fig 7D). Rotenone, on the other hand, had a less-robust pH-dependent effect where cells cultured at pH 8.5 were less susceptible with 80% viability (Fig 7D). Oligomycin toxicity was especially intriguing as this drug was the most toxic to PNEC cells at all extracellular pH values. At pH 6.5, there was only 27% viability in oligomycin-treated cells whose effect plateaued at pH 7.4 and 8.5 with 47% viability (Fig 7D). These findings further supported the OCR and ECAR data, demonstrating the reliance of PNEC cells cultured in acidic pH on OXPHOS for cell energetics and survival. Moreover, the highest and least pH-dependent toxicity seen with oligomycin further demonstrated the importance of complex V-derived ATP synthesis for PNEC cells cultured in all pH conditions, further supporting our finding that OCR was not directly affected by extracellular pH, and that PNEC cells cultured in physiologic and alkaline conditions could derive additional ATP from aerobic glycolysis relative to PNEC cells in acidic conditions.

### Synergistic effects of niclosamide and extracellular pH in PNEC cells

There is abundant data demonstrating that acidic extracellular pH enhances aggressive tumor features [10, 16, 42, 43]. Our data demonstrating that OXPHOS inhibitors could selectively target PNEC cancer cells in acidic pH led us to identify clinically-relevant pharmaceuticals that could synergize with acidic extracellular pH and thus target aggressive features of tumors. We searched the literature for pharmaceuticals that could inhibit mitochondrial function and identified the anti-helminthic drug niclosamide. Although many mechanisms of action have been attributed to this drug, one mechanism includes protonophoric activity or the ability to abolish the proton gradient in intracellular organelles [44–46]. Because this mechanism is similar to that of CCCP, we hypothesized that niclosamide could exert pH-dependent effects on mitochondrial potential and cell energetics in PNEC cells.

**Fig 8:**
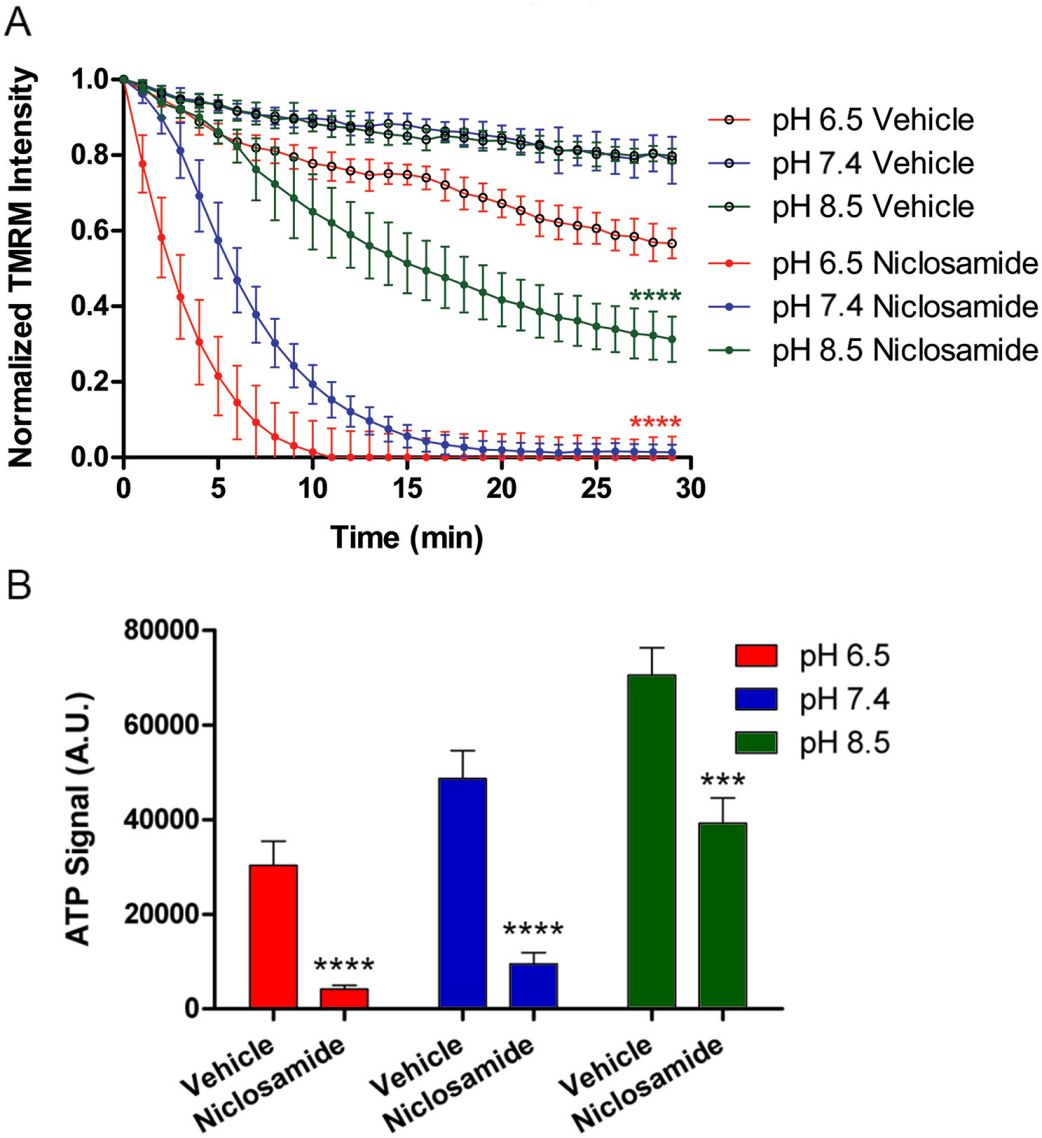
Niclosamide inhibition of mitochondrial function is enhanced with acidic pH in PNEC cells. (A) Kinetics of mitochondrial potential following addition of either vehicle (0.1% DMSO) or 10 μM niclosamide as a function of pH. (B) ATP levels in PNEC cells obtained 30 minutes following addition of either vehicle or 10 μM niclosamide. Significance calculated relative to pH 7.4 for panel A and relative to vehicle treatment for panel B. N = 10 samples per group for kinetics and N = 3 samples per group for ATP assay. ***p<0.001, ****p<0.0001. A.U.: arbitrary units.

First, we investigated the time and pH-dependent effects of niclosamide on mitochondrial potential. Following labeling with TMRM dye, we added 10 μM of niclosamide to cells at each respective extracellular pH. Time-resolved imaging of PNEC cells exposed to either DMSO vehicle or 10 μM niclosamide disclosed pH-dependent mitochondrial depolarization induced by niclosamide (Fig 8A). As expected, niclosamide had the most robust effects on depolarization at pH 6.5 (30% of initial potential at 5 minutes) versus pH 7.4 (69% initial potential at 5 minutes) versus pH 8.5 (90% of initial potential at 5 minutes). Interestingly, niclosamide had rapid effects on PNEC cells as evidenced by 100% depolarization within 12 minutes of niclosamide administration at pH 6.5 (Fig 8A).

Because mitochondrial depolarization abolishes the proton motive force required by the complex V ATP synthase to synthesize ATP, we investigated the effects of niclosamide on ATP levels in PNEC cells. Following addition of 10 μM niclosamide for 30 minutes at pH 6.5, 7.4, and 8.5, niclosamide treatment (relative to vehicle) resulted in significantly decreased ATP levels at all extracellular pH values. However, these effects were most pronounced at pH 6.5 where niclosamide treatment significantly reduced ATP levels by 86% compared to 80% at pH 7.4 and 44% at pH 8.5 (Fig 8B). These findings paralleled the mitochondrial complex inhibitor results and suggested that niclosamide treatment could be selectively toxic to PNEC cells at acidic pH. Moreover, given the dependence of cancer cells on nutrient consumption, glycolysis, and mitochondrial activity, we hypothesized that the pH-dependent metabolic effects seen in PNEC cells could be translated to other prostate cancer cell models.

### Synergizing extracellular pH and metabolic inhibition to enhance cell toxicity in PNEC and prostate adenocarcinoma cell lines

First, we determined if nutrient deprivation could synergize with physiologic and alkaline extracellular pH to enhance cell toxicity in other cancer cell models. In addition to PNEC cells, we used three human models for castrate-resistant prostate adenocarcinoma: C4-2B, PC-3, and PC-3M cell lines.

**Fig 9:**
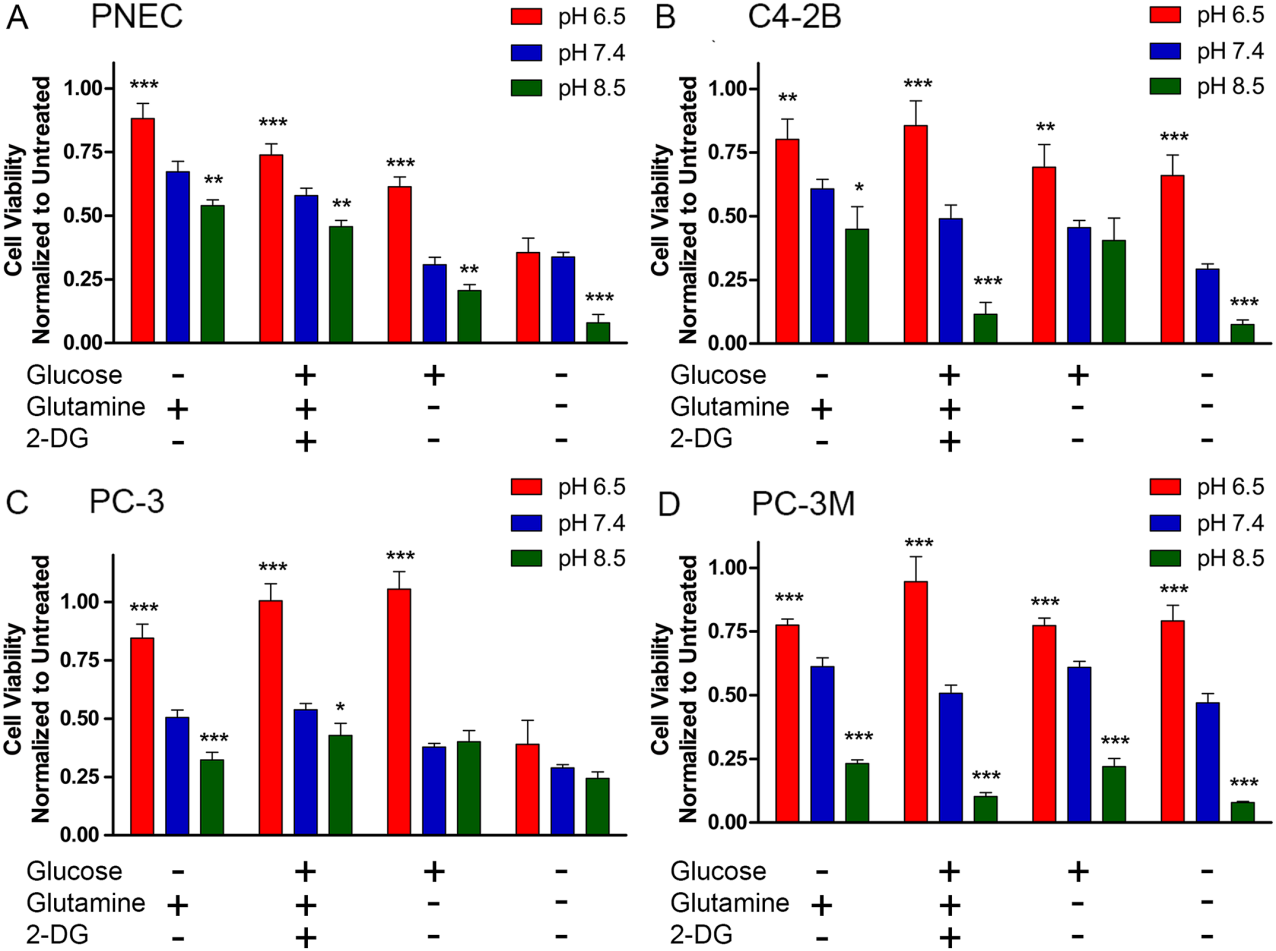
Nutrient deprivation has pH-dependent toxicity in castrate-resistant prostate cancer cell lines. Cell viability determined using the sulforhodamine B assay in (A) PNEC, (B) C4-2B, (C) PC-3, and (D) PC-3M prostate cancer cells demonstrate that inhibition of glucose and glutamine metabolism enhance cell toxicity with alkalinization. Statistical comparisons performed relative to pH 7.4 in each group. N = 4 samples per group. *p<0.05, **p>0.01, ***p<0.001. 2-DG: 2-deoxyglucose.

We cultured all four cell lines for two days under nutrient-defined conditions where two key nutrients glucose and glutamine were independently manipulated. We also tested the efficacy of 10 mM 2-DG (in the presence of 10 mM glucose) as an inhibitor of glycolysis to determine if chemical inhibition of metabolism could synergize with extracellular pH. We identified similar trends among all four cell lines where nutrient deprivation or glycolytic inhibition with 2-DG enhanced cell toxicity with alkalinization. In the case of PNEC cells, significant trends were identified with all treatment groups. PNEC cells incubated at pH 6.5 were the least susceptible to the toxic effects of glucose and glutamine deprivation and 2-DG treatment relative to other pH treatments. Conversely, PNEC cells cultured at pH 8.5 were more susceptible to the same treatments, further supporting our model (Fig 9A). Interestingly, glutamine-deprived PNEC cells were significantly less viable compared to glucose deprived PNEC cells at all three pH values and was most pronounced at pH 8.5 (54% viability in glucose-deprived media vs. 21% viability in glutamine-deprived media) (Fig 9A). Moreover, combined glucose and glutamine deprivation exerted the largest negative impact on PNEC cell viability and was also most pronounced in alkaline pH, resulting in 8% viability (Fig 9A). Together, these findings pointed to the potential importance of synergistic lethality of inhibiting both glucose and glutamine consumption in neuroendocrine cancer cells. Although glucose and glutamine deprivation had the greatest negative effect on PNEC cell viability in alkaline pH, there was also a significant decrease in viability at pH 6.5 in the absence of glucose and/or glutamine relative to cells cultured with both nutrients (Fig 9A). These findings suggest that both glucose and glutamine are required for cell viability in acidic pH, and at least in acidic pH, glucose may be engaged in non-lactate generating metabolism.

Similarly, the C4-2B cell line showed similar pH toxicity profiles. Statistically significant differences (relative to pH 7.4) were seen in all nutrient-deprived groups (Fig 9B). Notably, as with PNEC cells, combined glucose and glutamine deprivation with alkaline extracellular pH had the most potent synergistic effects with 7.5% viability (Fig 9B). These effects were seen in PC-3 and PC-3M cells as well. Although glutamine and combined glutamine and glucose-deprived PC-3 cells did not demonstrate significantly decreased viability at pH 8.5 relative to pH 7.4, there was a significantly robust difference between pH 6.5 and 7.4 in glutamine-deprived PC-3 cells (100% viability at pH 6.5 vs. 37.7% viability at pH 7.4) (Fig 9C). Although PC-3M cells are derived from a liver metastasis from its parental cell line, PC-3, we identified subtle differences in their toxicity profiles. pH-dependent toxicity from nutrient deprivation was more evident in PC-3M cells, with acidic pH conferring significantly-enhanced viability relative to pH 7.4 in all groups. As in PNEC and C4-2B groups, combined glucose and glutamine deprivation resulted in the greatest toxicity in PC-3M cells with 7.9% viability (Fig 9D). Together, these findings suggested: (i) the effects of extracellular pH and glucose and glutamine dependence could be translated across multiple castrate-resistant prostate cancer cell lines and (ii) mechanisms that increase intratumoral pH towards physiologic pH in concert with inhibition of glucose and/or glutamine consumption and metabolism could be used to enhance therapeutic efficacy.

**Fig 10:**
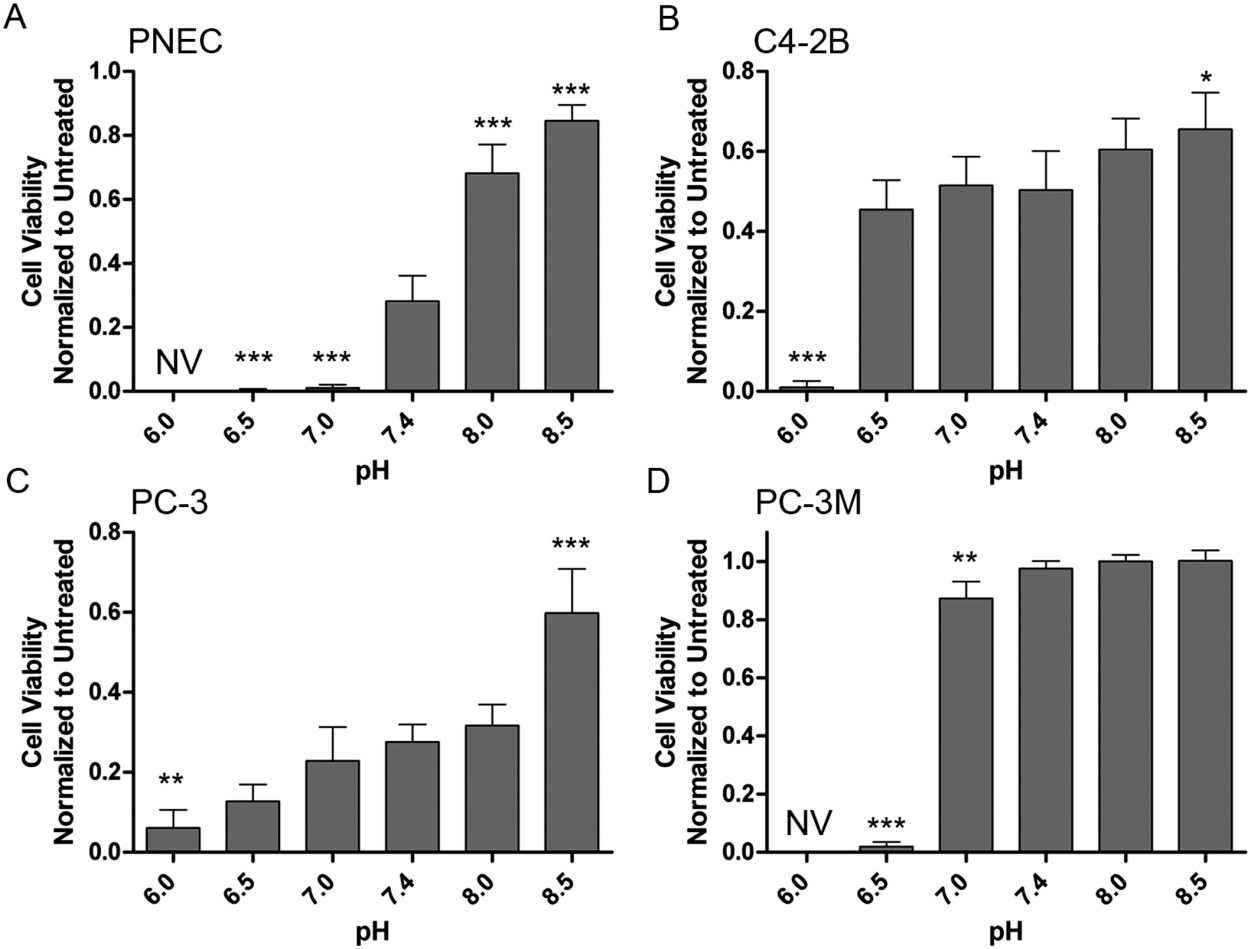
Niclosamide has pH-dependent toxicity in castrate-resistant prostate cancer cell lines. Cell viability determined using the sulforhodamine B assay in (A) PNEC, (B) C4-2B, (C) PC-3, and (D) PC-3M prostate cancer cells demonstrate that niclosamide enhances cell toxicity with acidification. Statistical comparisons performed relative to pH 7.4 in each group. N = 4 samples per group. *p<0.05, **p>0.01, ***p<0.001. NV: Untreated PNEC and PC-3M cells are not viable at pH 6.0.

Although there are current agents that have the ability to increase extracellular pH in tumors [47], we determined if niclosamide could be used to kill prostate cancer cells in acidic pH. Evaluation of niclosamide toxicity from pH 6.0 to 8.5 demonstrated significant effects on PNEC viability at acidic pH with only 0.2% viability at pH 6.5 (Fig 10A). Moreover, only 27.7% of PNEC cells were viable at physiologic pH (Fig 10A). Interestingly, PNEC cells were relatively resistant to niclosamide toxicity at pH 8.0 (68.2% viability) and pH 8.5 (82.6% viability). Vehicle-treated (and untreated) PNEC cells exposed to pH 6.0 were not viable (Fig 10A).

C4-2B and PC-3 cells showed a weaker pH-dependent response with significant toxicity relative to pH 7.4 seen only at pH 6.0. However, the overall range in viability across the pH spectrum of 6.0 to 8.5 in these two cell lines was not as robust as in PNEC cells. For example, C4-2B cells demonstrated only 84.6% viability at pH 8.5 (Fig 10B) and PC-3 cells demonstrated only 60% viability at pH 8.5 (Fig 10C). Interestingly, compared to the parental PC-3 cells, PC-3M cells had a much different pH-dependent niclosamide toxicity profile characterized by a relatively steep drop in toxicity at pH 6.5 (1.9% viability) from pH 7.0 (87.3% viability) (Fig 10D). Overall, the PC-3M response to extracellular pH was similar to that of PNEC cells characterized by (i) relative resistance to niclosamide toxicity at alkaline pH, (ii) significantly decreased viability at pH 6.5 and pH 7.0, and (iii) no viability at pH 6.0. These findings suggest the possibility that niclosamide could be used as an anti-cancer therapeutic that could selectively target the acidic extracellular environment in prostate cancer.

## Discussion

Multiple studies of the effects of normoxic acidosis on the phenotypes of various cancer cell models have demonstrated that acidity enhances cell invasion, a stem cell phenotype, resistance to immunotherapy, and resistance to chemotherapy [10, 12, 13, 42, 48]. Here, we have found that an acidic, normoxic environment can enhance susceptibility of cancer cells to OXPHOS inhibitors. This finding is potentially important, given that OXPHOS and mitochondrial function may enhance the energetics of therapy-resistant stem-like cancer cells and that acidity-induced conversion to an OXPHOS-dependent state may contribute at least in part to therapeutic resistance in tumors [49–52]. By developing a mechanistic understanding of the effects of extracellular acidity on PNEC cell metabolism, we have identified niclosamide as a potential drug that could be used to treat the acidic compartment of tumors that harbor cells with a malignant phenotype.

Our findings illustrate that acidic extracellular pH has direct effects on mitochondrial morphology and energetics. These findings are supported by recent evidence that mild extracellular acidosis (pH 6.5) can preserve ATP levels independent of oxygen levels in post-mitotic neurons. Moreover, acidosis restructures neuronal mitochondria, resulting in increased total length [53]. Although our data showed significantly lower ATP in PNEC cells cultured at pH 6.5 versus pH 7.4 and 8.5, PNEC cells compared to neurons are not post-mitotic and the lower ATP levels may represent a complex balance between enhanced ATP production from OXPHOS and enhanced ATP utilization in multiple pathways to maintain cell viability. In fact, extracellular acidification (independent of hypoxia) may specifically activate ATP-consuming metabolic pathways such as fatty acid synthesis mediated through fatty acid synthase (FAS) [54]. Thus, there may be specialized requirements for nutrients aside from glucose and glutamine required to power cellular metabolism at acidic pH in normoxia [55].

ATP synthesis driven by the proton motive force in mitochondria is governed by both mitochondrial membrane potential (ΔΨ_m_) and the proton chemical gradient (ΔpH_m_). Although ΔΨ_m_ contributes most of the proton motive force, ΔpH_m_ drives flux of substrates needed for respiration [56]. ΔΨ_m_ has also been used as a measure of mitochondrial fuel availability where ΔΨ_m_ declines with nutrient withdrawal, conversely increases with nutrient supplementation, and is also positively regulated by Akt/mTOR signaling [57–59]. Our data demonstrating that increasing extracellular pH also enhances ΔΨ_m_ supports these previous findings. Conversely, acidification-induced decrease in glucose and glutamine consumption as well as ΔΨ_m_ also fits the current model where cancer cells cultured under acidic conditions maintain an autophagic state that can similarly be induced from growth factor and nutrient deprivation [60, 61]. However, it is still not known if the change in extracellular pH directly affects ΔΨ_m_ and thus the proton motive force. Previous evidence suggests that intracellular pH is highly buffered and the effects of extracellular acidification are not mediated by intracellular acidification in T lymphocytes [62]. It remains possible that the effects of extracellular pH on cancer cell metabolism may be from cell signaling pathways involving pH sensors including the G-coupled protein receptor T cell-associated gene 8 (TDAG8) that has the ability to modulate metabolic drivers such as MYC [63, 64].

The impact of extracellular alkalinization on cancer metabolism is an intriguing one that is relevant to therapeutic development, including the popular use of sodium bicarbonate as an agent to alkalinize the tumor microenvironment [16, 65, 66]. However, the ability to alkalinize the tumor microenvironment, at least with bicarbonate, may be mathematically challenging [16, 65]. Thus, the ability to therapeutically reach pH 8.5 in the tumor microenvironment may be unachievable. However, our data suggest that elevation of extracellular pH to at least physiologic levels (pH = 7.4) may be sufficient to take advantage of therapeutic approaches that target nutrient consumption pathways. This paradigm would not only be relevant to bicarbonate-based therapies, but novel agents such as calcium carbonate nanoparticles (nano-CaCO_3_) that have the ability to neutralize tumor pH to physiologic levels and reduce tumor growth [47]. Our data suggest that the anti-tumor activity of alkalinization may be a secondary effect that results from enhancing nutrient consumption in tumor cells that are in an environment where nutrients are already scarce, thus enhancing cancer cell death.

However, the presence of both genotypic and phenotypic heterogeneity within tumors is hypothesized to give rise to malignancy and treatment resistance, and thus proposes why single targeted therapy does not work [67]. Our data suggest that inhibitors that target both OXPHOS and glycolysis could have an additive effect in reducing tumor burden in both metabolic compartments. In fact, there is evidence that starvation in combination with metformin, a complex I OXPHOS inhibitor, can significantly reduce tumor growth in colon and breast cancer models [68]. Because tumors can develop resistance mechanisms to chemotherapeutics including metformin the identification of drugs that target other components of critical pathways such as OXPHOS are urgently needed [69, 70]. For example, the anti-helminthic niclosamide has been applied as an anti-tumor agent to a variety of cancers, including castrate-resistant prostate cancer androgen receptor variants [44, 71, 72]. Our data suggest that niclosamide, in combination with other pharmaceuticals or even diets that modulate metabolic pathway flux such as glycolysis could have an additive effect on cancer therapy.

We demonstrated that niclosamide can depolarize mitochondria and deplete ATP within minutes of addition to cells in a pH dependent manner. These findings potentially correlate with previously described actions of niclosamide that demonstrate disruption of pH homeostasis in flukes, cytoplasmic acidification with dissipation of proton gradients across intracellular organelles, and suppression of acidic lysosome function and trafficking [73–77]. Thus, the protonophoric activity of niclosamide is expected to be inversely proportional to the extracellular pH and is therefore more toxic in acidic pH. Furthermore, the pH-dependent effects of niclosamide and the acidic environment of tumors relative to physiologic pH may allow some degree of tumor specificity and reduced toxic side effects. This is further supported by in vivo studies of niclosamide that demonstrate that oral administration is sufficient to induce a mild mitochondrial uncoupling that can reverse diabetic symptoms in mice [45]. The more robust differences in niclosamide activity seen in PNEC and PC-3M cell lines in this study suggest a possible correlation with the inability of these cells to maintain viability in extreme acid stress (pH 6.0). Moreover, there may also be implications in how the glycolytic capacity of the cancer cell impacts its ability to survive extreme acidic extracellular pH, as PNEC cells demonstrate weak acidification of media under conventional culture conditions.

Although an extracellular pH of 8.5 may be considered solely an “artificial” means of investigating metabolism in an *in vitro* system, it should be noted that this could be a physiologically relevant system for studying cancer metabolism. Bone (a common site for metastasis from prostate and many other cancers) is alkaline where osteoblast activity has been reported to peak around an extracellular pH~8 and whose extracellular alkaline phosphatases required for bone formation have peak activity around pH~9 [78–80]. Therefore, the development of tools that facilitate the investigation of cancer cell interactions with these alkaline matrices and the development of agents that can effectively measure the pH of these environments are necessary.
